# Voxel-based morphometry with templates and validation in a mouse model of Huntington’s disease^☆^

**DOI:** 10.1016/j.mri.2013.06.001

**Published:** 2013-11

**Authors:** Stephen J. Sawiak, Nigel I. Wood, Guy B. Williams, A. Jennifer Morton, T. Adrian Carpenter

**Affiliations:** a Wolfson Brain Imaging Centre, University of Cambridge, Box 65 Addenbrooke’s Hospital, Cambridge, CB2 0QQ, UK; b Behavioural and Clinical Neuroscience Institute, Department of Experimental Psychology, Cambridge, CB2 3EB, UK; c Department of Pharmacology, University of Cambridge, CB2 1PD, UK

**Keywords:** Automated analysis, Translational methods, Image processing, Huntington’s disease, R6/2 mouse

## Abstract

Despite widespread application to human imaging, voxel-based morphometry (VBM), where images are compared following grey matter (GM) segmentation, is seldom used in mice. Here VBM is performed for the R6/2 model of Huntington’s disease, a progressive neurological disorder. This article discusses issues in translating the methods to mice and shows that its statistical basis is sound in mice as it is in human studies. Whole brain images from live transgenic and control mice are segmented into GM maps after processing and compared to produce statistical parametric maps of likely differences. To assess whether false positives were likely to occur, a large cohort of ex vivo magnetic resonance brain images were sampled with permutation testing. Differences were seen particularly in the striatum and cortex, in line with studies performed ex vivo and as seen in human patients. In validation, the rate of false positives is as expected and these have no discernible distribution through the brain. The study shows that VBM successfully detects differences in the Huntington’s disease mouse brain. The method is rapid compared to manual delineation and reliable. The templates created here for the mouse brain are freely released for other users in addition to an open-source software toolbox for performing mouse VBM.

## 1 Introduction

Huntington’s disease (HD) is an inherited neurological condition characterised by chorea, progressive cognitive deficits and behavioural changes [1]. No cure has yet been found and the condition is invariably fatal. The discovery that the disease results from a single defective gene has led to the development of several transgenic animal models in mice [2], [3], [4] of which the most widely used in HD research is the R6/2 mouse which contains a fragment of the gene responsible for the disease in humans [5].

MRI studies were first used simply to identify in vivo the characteristic features already known from post mortem studies of established HD, such as the enlarged lateral ventricles seen with atrophy of the caudate nucleus [6]. The development of sophisticated automated image analysis algorithms, however, has led to a wide number of studies conducted to identify pathology across the brain in established HD but more importantly in preclinical patients, before symptoms are apparent [7], [8], [9], [10], [11], [12]. The most useful animal models will recapitulate features seen in human patients, and this includes an MRI phenotype. In the R6/2 mouse, we have previously demonstrated patterns of atrophy of structures known to be affected in humans [13], [14].

R6/2 mice with a CAG repeat length of 250 (as used in the present study) have brain pathology characterised by abnormal aggregates of protein [15] and changes in synaptic plasticity [16] by three weeks of age. They show progressively impaired motor [17] and cognitive [18] function from around 6 weeks of age. The mice stop growing around 10-12 weeks of age and then start to lose weight. They typically die prematurely at around 24 weeks of age [17]. These mice have also been shown to have disintegrated circardian rhythms [19] and cardiac dysfunction [20].

Voxel-based morphometry (VBM) describes the automated process of analysing morphological differences between images of brains by performing voxel-wise statistics [21]. Images are registered into the same stereotactic space and segmented into images of tissue classes, with four commonly represented: grey matter (GM), white matter (WM) and cerebrospinal fluid (CSF) and ‘everything else’.

Typically the GM segmented image will form the basis of statistical tests used to assess hypotheses concerning the data in a statistical parametric map (SPM) which is then thresholded to identify localised regions where (e.g.) a null hypothesis can be rejected at a particular level.

Since its introduction in 1995 [22], the technique has seen rapidly growing usage: a simple keyword search for the technique on the PubMed repository (http://ncbi.nlm.nih.gov) reveals 260 papers from 2000-2005, 1,152 from 2006 to 2010 and 454 papers in 2011 alone. The technique can be widely applied to studies in neurology, psychiatry and psychology to identify structural differences in healthy as well as abnormal brains.

Despite this, the method has seen little application to the mouse brain. Since VBM is still the most common analysis technique used for human brain morphometry, it is surprising that more groups performing translational research with mouse brain imaging are not employing it. We speculate that the reasons for its lack of use are the perceived complexity of the method coupled with the lack of software tailored to non-human brains. After considerable development for human use [23], [24], [25], [26] the major software packages used, SPM (Wellcome Trust Centre for Neuroimaging, University College London, UK) and FSL (FMRIB, University of Oxford), are pre-configured with default settings that do not work for non-human brains. Simplified interfaces that have led to the huge success of these packages in human studies, used in the majority of papers cited above, are obstructive for other species as they conceal many important scaling parameters. In an effort to boost the usage of VBM in the mouse brain, we have released a software toolbox for SPM (SPMMouse) including our templates so that mouse brain analysis can be performed as readily as human studies.

In this study, we apply VBM as described to in vivo images of the R6/2 transgenic mouse brain with wildtype (WT) controls. To our knowledge this is the first application of VBM as described to in vivo data from a HD mouse model.

To establish whether the statistical basis of this method is sound, we took a larger cohort of brains from an open-access library of ex vivo mouse brain images. We randomly permuted labels of these datasets as WT or transgenic to see how often the method produced false positive results compared to predictions by chance. Additionally, we kept track of where these false positive results were discovered so that we could identify whether type I errors in this technique are biased towards particular brain regions. A larger pool of datasets was used for this so that we were more likely to identify subtle effects.

We have included in the methods section below a brief discussion of the purpose of each step in the VBM pipeline and our rationale for selecting the parameters we have used so that readers unfamiliar with the VBM process can understand the choices that we have made.

## 2 Methods

### 2.1 Image acquisition

#### 2.2 Animals (in vivo)

Six WT control mice (aged 14±1 weeks), and six R6/2 transgenic mice with 250 abnormal CAG repeats (age 15±1 weeks) were imaged for this study. There was no significant difference in age between groups and at this age a phenotype is clearly established. To put this age into context more than 95% of this line of R6/2 mice will die as a consequence of having this gene by 20 weeks of age [17].

Animals were anaesthetised with isoflurane (1–2% in 1l/min O_2_). Respiration rate was monitored using a respiratory pillow to control anaesthetic depth (SA Instruments Inc., Stony Brook, NY, USA). Core body temperature was measured with a rectal probe and maintained in the normal range using a flowing water heated blanket.

All procedures were approved by a local ethical review committee and were performed in accordance with the UK Animals (Scientific Procedures) Act 1987.

#### 2.2.1 Animals (ex vivo)

For the statistical validation, ex vivo images were taken from the Cambridge HD public library [27]. The brain images used were from mice aged 18 weeks of mixed sexes with 42 WT brains and 42 R6/2 mice with 250 CAG repeats used and the animals originate from the same colony as the in vivo mice used here.

#### 2.2.2 Imaging parameters

Images were acquired at 4.7 T in vivo using a rapid-acquisition with relaxation enhancement (RARE) sequence (TR/TE_eff_ 3500/32ms, ETL 16 FOV 25.6×19.2×10.0mm^3^, matrix 256×192×100, spatial resolution 100μm in 1h33m). Images were acquired and reconstructed using ParaVision 4.0 with a Bruker BioSpec 47/40 system (Bruker Inc., Ettlingen, Germany). An actively decoupled quadrature-mode mouse brain surface coil (model T9788) was used for signal reception and a 72-mm birdcage coil (model T5346) was used for transmission, both supplied by Bruker.

The ex vivo protocol is fully described elsewhere [27], a RARE sequence is also used though higher resolution (70 μm isotropic) and 4 averages were used for greater signal to noise ratio.

### 2.3 Pre-processing

**Fig. 1:**
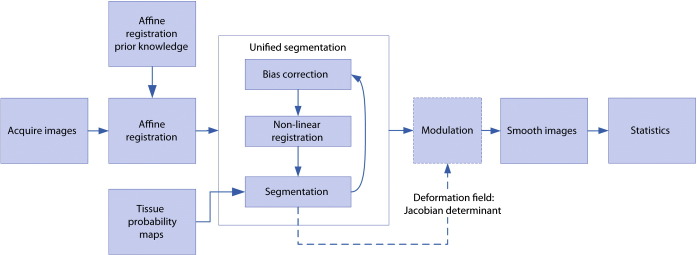
Steps involved in a voxel-based morphometry analysis.

The steps involved in VBM are illustrated in Fig. 1 and discussed in more detail below.

Before voxelwise statistics can be calculated, images have to be registered into the same stereotactic space and segmented to give tissue probabilities. Affine registration refers to a global geometric transformation applied identically to each part of the image which preserves parallel lines. Non-linear registration provides a finer match between images by allowing local transformations that adjust different parts of the image in different ways. Segmentation based on intensities is unreliable without taking account of coil inhomogeneity [28], and registration is usually addressed with first an affine step followed by a finer non-linear step. These are considered in more detail below.

#### 2.3.1 Affine registration

For human studies, Tailarach or MNI coordinates are often used for the analysis and presentation of results, see e.g. [29]. The SPM software is supplied with templates and atlases in MNI space. For rodent studies, the most common coordinates used are those of the Franklin and Paxinos atlas, based on the bregma point of the skull [30]. For the mouse brain, we calculated the distribution of affine parameters from a large number of ex vivo scans [13] and this is included in the SPMMouse package. Taking advantage of this prior knowledge has been shown to improve the quality of affine registration [31]. In addition, the matrices encoded in these headers are aligned with the coordinates from the Franklin and Paxinos atlas for easy reference to stereotactic space when reading and reporting results from the software. The mouse brain templates included in SPMMouse for initial alignment and/or registration were derived as a minimum-deformation atlas [32] in our previously published VBM study. The mean and standard deviation of the rigid transformation invariant parameters (i.e. scales and shearing values) in the polar decomposition form used by SPM were calculated and stored as a mean and covariance matrix for use in SPM.

The mean and standard deviation of the affine parameters of the brains to the atlas is 1.00±0.01, 1.00±0.02, 1.00±0.02 for scaling in left-right (*x*), anterior-posterior (*y*), inferior-superior (*z*) directions and 0.00±0.02, 0.01±0.04, 0.02±0.01, for shearing matrix values *xy*, *xz* and *yz*. In comparison with the human brain priors for MNI templates in SPM based on 227 scans, these values are about a quarter as large (the human standard deviations are 3–4% for each axis).

#### 2.3.2 Non-linear registration and segmentation

The registration step used routinely in SPM uses approximately 1,000 parameters. These are enough to correct for overall brain shape but overall this method is outperformed by algorithms employing more parameters [33]. Most approaches to VBM consider the brain to consist of 4 classes, namely GM, WM, CSF and ‘everything else’. SPM models each voxel as a weighted sum of each tissue type based on a mixture of (typically two) Gaussian distributions. This helps deal with partial volume effects which confound the segmentation process. The unified segmentation model of SPM5/8 estimates parameters for RF inhomogeneity, non-linear registration and segmentation iteratively [23].

Within SPM, it is possible to use more tissue classes for modelling the brain and more parameters to represent the warp using ‘New Segment’ based on DARTEL [34]. This open-source registration code is scale invariant in its implementation, independent of voxel size for its regularisation parameters when existing parameters are used. For generating new templates, however, the ‘smoothing parameters’ are based on millimetre-scale parameters which are scaled to the fourth power internally to calculate bending energy. The SPMMouse interface allows templates to be created with smoothing parameters commensurate with the scale differences between human and mouse brains so that these more advanced algorithms can be used readily with the mouse brain.

Surface coils are often used to image the mouse brain due to improved filling factor and higher signal-to-noise ratio (SNR). We found the RF non-uniformity modelling in SPM works well for mouse brain images acquired with solenoid and surface coils when an appropriate spatial cutoff (10-15mm) was used [23].

#### 2.3.3 Image smoothing and modulation

If parameters encoding morphological difference are considered as a signal that can be obtained amongst the noise of a random field, then the matched filter theorem suggests the optimal spatial smoothing kernel has the same scale as the signal to be found (see e.g. [35]). In addition, smoothing confers benefits on the normality of the statistics and reduces the impact of misregistration between images. An isotropic smoothing kernel of 5–10 times the smallest voxel dimension is typical of studies in the literature. Excessive smoothing will tend to increase power at the expense of spatial localisation.

For the in vivo study here, an isotropic smoothing kernel of 400 μm was used.

### 2.4 Statistics

Following pre-processing, structural measures are modelled in a general linear model (GLM) to test hypotheses. An appropriate model for the R6/2 mouse study here is a two-sample Student’s *t*-test between groups. A good overview of modelling brain imaging data with GLMs can be found in [36].

The number of voxels compared for mouse brain images is similar to the human brain case and such mass univariate questions are vulnerable to type I errors without appropriate treatment of multiple comparisons. The main methods used to address this problem are to control the family-wise error (FWE) or false-discovery rate (FDR). For a given threshold α, the FWE will on average contain at least one voxel that is a type I error with probability α [37]. The FDR control is less conservative and will, on average, report a proportion α of type I errors amongst the results which survive the threshold [38]. This corrected *p*-value can be computed amongst voxels or clusters (‘topological-FDR’, see [39]).

An alternative to height-thresholding is to use cluster extent thresholding, to accept only those clusters comprising a critical number of voxels [40]. It has been demonstrated, however, that cluster extent thresholding alone does not always offer sufficient control of type I errors in VBM in the human brain [21]. Efforts have also been made to combine both techniques, such as the threshold-free cluster enhancement method, which uses both the extent and height to derive an adjusted value, which then must be compared to a null distribution via permutation testing to establish significance [41].

For the present study, a voxelwise FDR correction *q* < 0.05 was used with no extent threshold applied. From previous work and knowledge of the mouse model, reductions in GM would be expected, so a one-tailed *t*-contrast was used to compare mean GM between WT and R6/2 mice. To correct for overall brain volume, total intracranial volume was used as a covariate that was allowed to interact with genotype.

### 2.5 Statistical validation

The main goal of the validation here was to investigate type I errors in VBM for the mouse brain. Led by the widely used rationale for VBM in the human brain [21], here we first assess the normality of residuals for the validity of parametric testing and secondly we investigate both the number and distribution of false positives through the brain when the null hypothesis is true.

In simple terms, the validation aims to show that the differences revealed by this method can be relied upon as genuine. This is done by taking random groups of brains and artificially labelling them as being control or transgenic brains then applying the technique to find differences between the groups. As the groups are artificial, there should not be any significant differences. If the statistical treatment is appropriate, the false positives found in this method should appear at a known rate (e.g. for FWE p < 0.05, 5% of such studies should contain at least one false positive voxel). Comparing the false positives found against those expected shows whether the statistical framework is satisfactory, particularly if (as is known to be the case frequently in human studies) the residuals from the linear model deviate from a normal distribution.

In addition, all of the statistical parametric maps from each study were kept so that counts could be made of voxels where false positives were found so that we could identify whether particular regions in the brain were more susceptible to them.

It is easier to find evidence of deviant statistics amongst larger datasets than smaller datasets, so the data of our previous ex vivo study were used to probe whether the assumptions commonly held in VBM hold for mouse brain studies. It seemed unlikely that the twelve in vivo brains used in the present work would be sufficient for a convincing demonstration of deviation from Gaussian behaviour or to show that false positives are biased to particular brain regions, so the larger cohort of ex vivo brains was used. All of these data have been made available freely online and full details of their acquisition parameters have been published previously [27].

#### 2.5.1 Normality of residuals

Analysis of human imaging data showed that although there was a deviation from normality in the distribution of residuals, this did not have any significant effect on the expected number of false positives or their distribution throughout the brain.

Taking the design matrix from our previously-published VBM study [13], a voxel-based comparison of residuals was performed. QQ-plots were used to assess normality, specifically by taking the correlation coefficient between the sorted *j*th of *J* residuals and the quantile values given by

(1) $$q_{j}=\sqrt{2}\;\text{erfinv}\left(2\cdot \frac{j-\frac{3}{8}}{j+\frac{1}{4}}-1\right)$$

where erfinv is the inverse error function.

For comparison, coefficients were calculated for data drawn from a Gaussian distribution the same size of the image data repeated sufficient times for relative bin populations to change below 0.1%. All of the calculations were performed with Matlab 7 (Mathworks, Inc.).

#### 2.5.2 Type I error

To examine the type I error rate seen in the mouse VBM study, the VBM analysis was repeated 2,048 times with random group permutations. The number of permutations is arbitrary, but the larger it is the more likely deviant results are to be detected. We chose 2,048 for computational efficiency in running the VBM process on a cluster of 64 machines with 32 processes per machine. Specifically, 42 subjects from the control group and 42 subjects from the disease model group were entered into a design with randomly assigned labels of genotype. Total intracranial volume values for each subject were also entered as a covariate. SPM maps were produced for each of the 2,048 random designs. A threshold was applied to the maps based on the calculated family-wise error (FWE) height threshold cut off based on random field theory (RFT) implemented in SPM5 corresponding to a corrected *p*-value of 0.05. The contrast used was an *F-*contrast [− 1 1 0] to test two-tailed differences between GM probabilities based on genotypes.

We also compared the *F*-scores calculated in the permutation analysis with those expected under the null hypothesis and examined the distribution of false positives without correction for multiple comparisons to determine whether the technique is biased to particular brain regions.

## 3 Results

### 3.1 Pre-processing

**Fig. 2:**
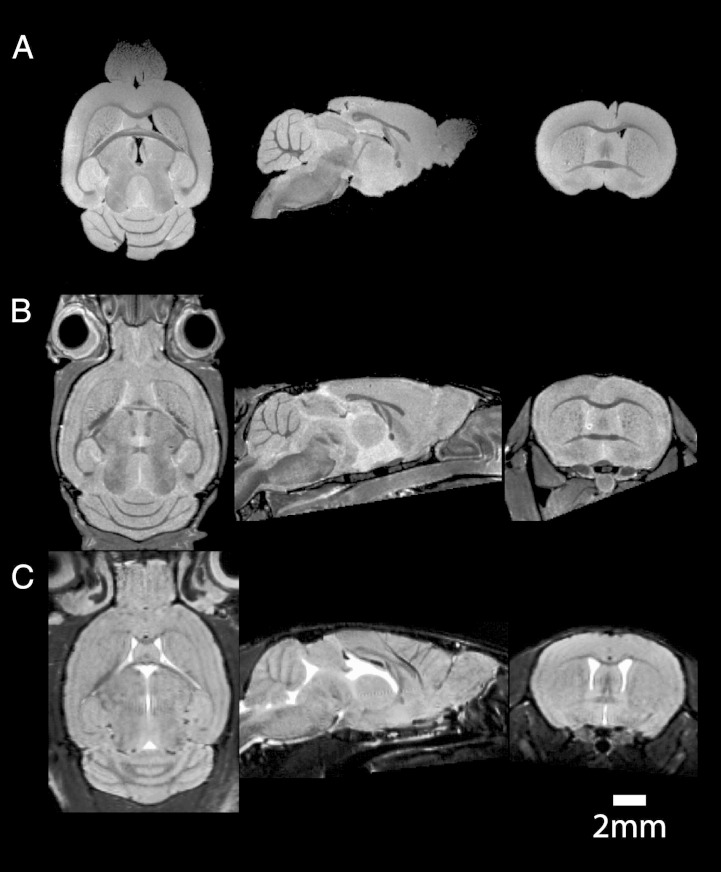
High resolution mouse brain images acquired A: ex vivo, extracted from skull; B: ex vivo, skull intact and C: in vivo. Note that GM/WM contrast is greater in fixed tissues and the skull-intact brain shape corresponds more closely to the in vivo images.

To aid comparisons with other studies, Fig. 2 shows how images from extracted brains (A) compare with intact ex vivo head scans (B) and in vivo (C) scans.

**Fig. 3:**
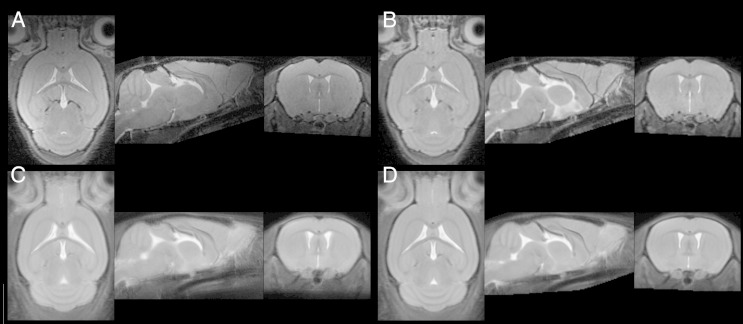
A: A typical in vivo mouse brain image before processing. B: the same image after bias-correction and affine transformation to the template. C: shows the average of 12 images after affine and non-linear registration using basis functions (512 parameters) and D: shows the average of DARTEL-transformed images (~ 106 parameters).

Fig. 3 shows a typical image before processing (A) and after bias-correction and affine registration (B). Panels C and D show average images of the 12 in vivo datasets after non-linear registration with basis functions (512 parameters) and DARTEL registration (10^6^ parameters) respectively. The sharper image seen from the DARTEL average shows that better voxel-wise correspondence has been achieved across datasets for the homologous anatomical features.

**Fig. 4:**
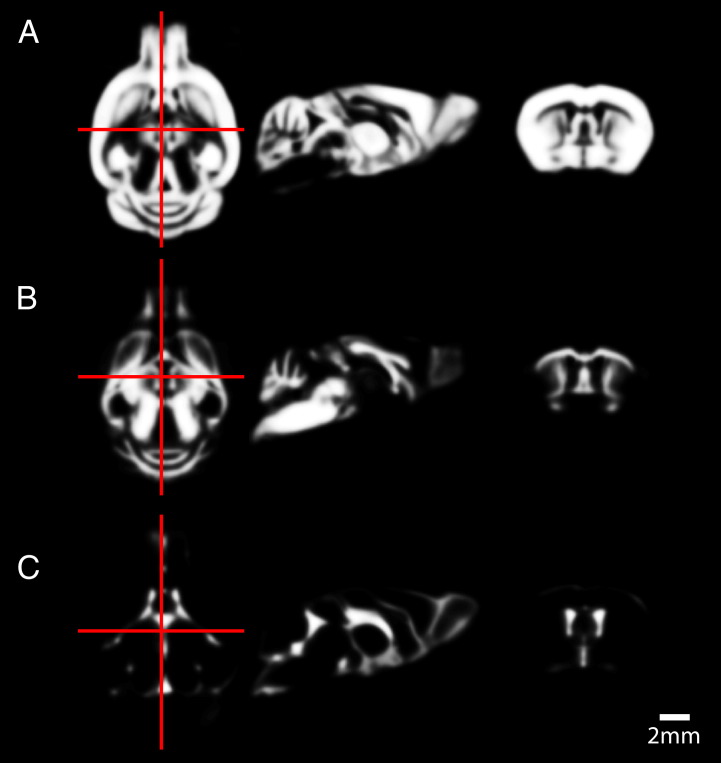
Tissue probability maps for A: GM, B: WM and C: CSF in the living mouse brain.

Tissue probability maps from the R6/2 mouse HD study are included in SPMMouse for the analysis of mouse brain data and are shown in Fig. 4.

### 3.2 Statistical validation

**Fig. 5:**
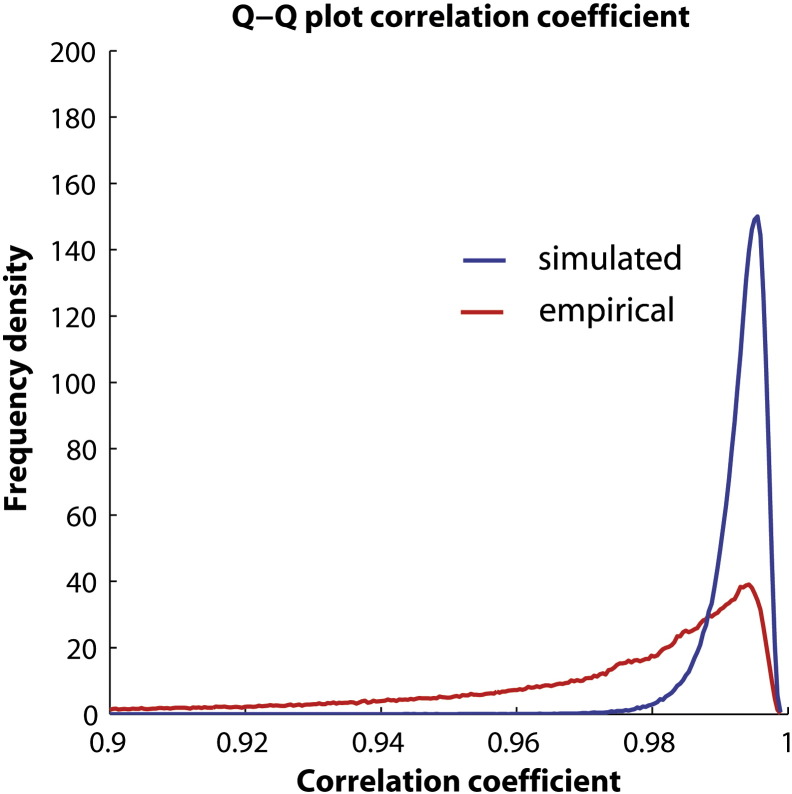
QQ-plot coefficients found by assessing empirical residuals from the HD study matrix (89 brains of 106 voxels) compared against those obtained from simulated Gaussian data.

Fig. 5 shows the distribution of *QQ*-plot correlation coefficients compared between the empirical VBM data from the ex vivo brains and the simulated Gaussian data. Although the results follow the trend of simulated Gaussian data, clear discrepancies can be seen between the two.

Of the 2,048 permutation analyses, 2,040 showed no significant voxels. Of the eight analyses yielding some positive results, the largest SPMs showed 21 and 31 voxels in 3 and 1 clusters respectively, compared to 8,192 voxels in 31 clusters with the real design matrix.

**Fig. 6:**
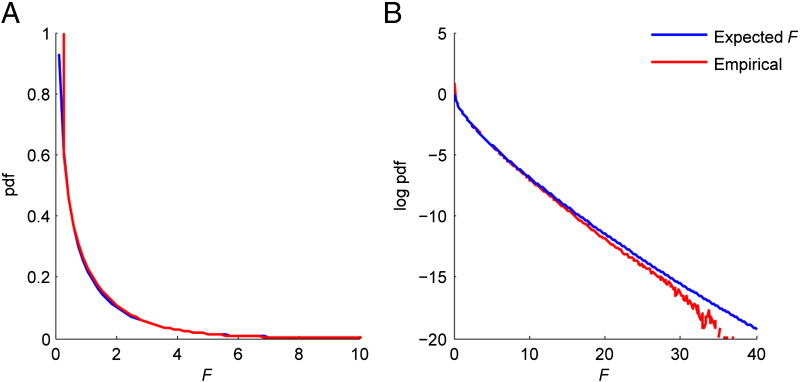
Comparison of the expected *F* statistic pdf and the empirical *F* values obtained in 2,048 whole brain SPMs where the null hypothesis should prevail on regular (panel A) and log (panel B) scales. Deviation at larger *F* values is due to low statistics (e.g. 99.9% of voxels have *F* < 11.1).

Fig. 6A shows a cumulative plot of the *F*-statistic probability density function calculated in the 2,048 trials with the expected *F* shown for comparison, with Fig. 6B showing the same on a log scale. The experimental data show good agreement with the simulation.

**Fig. 7:**
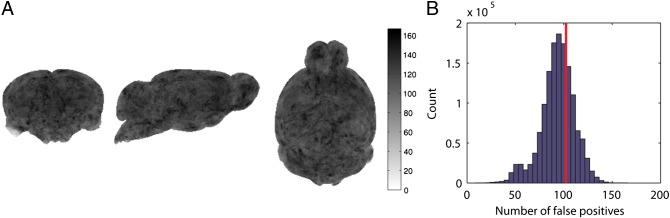
A: Maximum intensity projections (in coronal, sagittal and horizontal views) of false positive counts in 2,048 permutations of a design matrix thresholded at p < 0.05 uncorrected. B: Histogram showing counts over voxels, solid line indicates expected number of false positives.

An uncorrected threshold at significance level *p* < 0.05 corresponds to *F* > 3.9 for these data. Fig. 7A shows the distribution of voxels above this threshold counted over the whole brain as a maximum intensity projection in three orthogonal sections. Fig. 7B shows the histogram of significant counts for each voxel. For 2,048 tests at 5% significance the expected number is 102, this is shown as a solid line on the figure.

### 3.3 In vivo R6/2 mouse differences

**Fig. 8:**
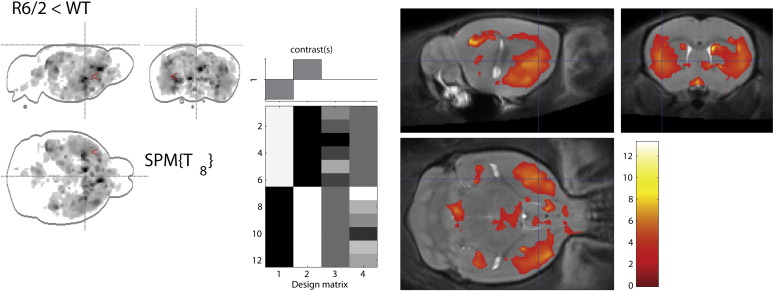
Presentation of the VBM results produced by the SPM software with the SPMMouse plugin. From left to right, a maximum-intensity projection (MIP) of the results is shown in sagittal, coronal and horizontal sections; the design matrix for the study; and a coloured overlay showing the location of significant clusters. The colour bar refers to Student’s t-score with 8 degrees of freedom. Values shown indicate GM scores lower R6/2 mouse compared to WT control mice (p < 0.05, voxelwise FDR-corrected).

Fig. 8 shows regions where GM values were significantly lower in the six R6/2 mice than in the WT controls. As expected, and in line with the ex vivo study, the striatum features prominently in the findings but there are also changes in somatosensory cortex and the olfactory bulbs. Differences are also seen in the amygdala and hippocampal formation in addition to frontal and parietal cortex. The pattern of findings reflect widespread atrophy, with reduced GM around each ventricle, in particular ventricles III and IV, and markedly in the periaqueductal grey matter (PAG) surrounding the aqueduct.

Using a more conservative treatment for multiple comparisons (FWE *p* < 0.05) revealed no significant voxels, but on a cluster level there were 2 significant clusters centred in the striatum and PAG (Paxinos coordinates bregma (− 0.7, 0.1, − 3.6mm) and (− 0.5, − 5.3, − 3.2 mm), respectively).

## 4 Discussion

We have shown that VBM can be readily performed in the mouse brain using the same software that is used for human studies with our templates. Once the data are retrieved from the scanner, the entire VBM process from initial alignment of subject data to reviewing statistical parametric maps takes 1–2 hours depending on the number of subjects. Manual methods such as region of interest (ROI) delineation typically take multiple hours per subject depending on the number of ROIs involved.

In VBM, morphology is examined across the entire brain without need of a prior hypothesis. Where such hypotheses exist they can be used to reduce the burden of multiple comparisons, e.g. by masking the data appropriately.

In this article we have added to our previous work by showing differences between WT and R6/2 brain images acquired in vivo. Unlike manual morphometry or histological analysis, user input to this methodology is minimal (required only for initial alignment of brains prior to the local optimisation of registration parameters), reducing operator bias.

From our experience in practice, and a regular point of discussion on the SPM mailing list, the most common area of failure for VBM pre-processing occurs in the affine registration step. If it presents problems, the step should be controlled by less liberal regularisation or where a good manual starting point is used it can be omitted entirely with good results.

Our previous morphological study [13], [14] examined mice close to the end-stage of disease (18 weeks) but here we have shown that many of the changes reported are in fact present 4 weeks earlier. This fits well with behavioural measures from similar mice in our facility: R6/2 mice with 250 CAG repeats start losing body weight at 10–12 weeks, and show significantly reduced grip strength and rotarod performance [14]. A difference from our previous study is that there were no findings in the cerebellum seen in these mice. We cannot say whether this is due to the lower power due to the smaller group sizes here or if these changes are only present in a later stage of disease. There is evidence however that CAG instability, characteristic of polyglutamine expansion disorders such as HD, is less marked in the cerebellum compared to other brain regions (e.g. neocortex and striatum) in both HD patients and the R6/2 mouse [42] so atrophy in these regions would be less marked at 14 weeks explaining why it was not found here.

The statistical analysis shows that despite the deviation from normality seen in the distribution of the residuals, the number of false positives per voxel does not deviate from that expected and the spatial distribution of false positives across the brain is not biased to particular structures. If the false positives were more likely to occur in particular brain regions then Fig. 8a would not have such a homogeneous appearance, but rather would show hyperintensity in the regions where the false positives occurred. Fig. 8b shows that the number of false positives is close to the expected values, but falls slightly below the expected line. This indicates that the FWE control rate is slightly too conservative, but the discrepancy here is negligible for practical purposes.

As discussed previously, alternative schemes are available for controlling the type I error rate such as the FDR [38].

### 4.1 Criticisms of the VBM approach

An obvious weakness of voxel-based approaches is their dependence on the assumptions that voxels represent homologous anatomy between subjects for a sensible comparison. The major criticisms in this regard were led by Bookstein [24]. The objection is relevant if segmented images are binarised, as with perfect registration of images (in the sense that source images appear identical to the target when thresholded) there will be no residual differences for a statistical comparison. Where segmented images actually represent probabilities of class membership however, and quantities are conserved by multiplying by the Jacobian determinants (‘modulation’ or ‘optimised-VBM’), the objection is much less important as shape differences in the original images form part of the statistic which is tested for analysis.

Improvements to VBM since its introduction (most notably incorporation of Jacobian determinants) do not entirely eliminate these concerns, and investigators seeking to interpret their findings must always consider other possibilities for the rejection of the null hypotheses apart from neuroanatomical differences in images. These possibilities can include systematic differences in images (e.g. differences in subject motion between controls and patients, whether control subjects have the same positioning in the scanner as other subjects, etc.).

**Fig. 9:**
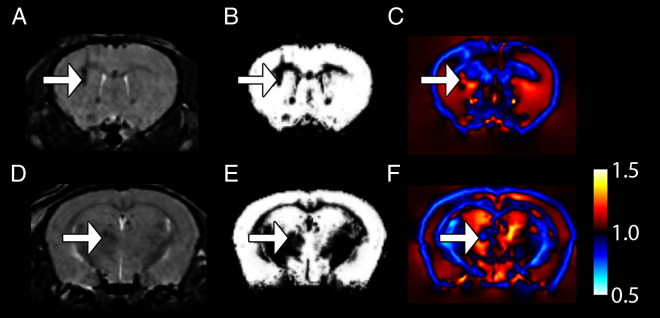
showing heterogeneity of Jacobian determinants in hypointense lesions. Sections here are taken from the same brain image at the level of the anterodorsal caudate putamen (A-C) and the thalamus (D-F). These images show that hypointense lesions in each region (arrows) are reflected by reductions in GM values (B, E) but heterogeneous changes in the Jacobian determinant (C, F). Colour scale refers to Jacobian determinant.

VBM has already shown to provide information complementary to that found by manual ROI-based methods and image registration methods such as DBM/TBM, and forms part of the arsenal of techniques available to exploit the richness of the datasets from the MRI scanner. The majority of studies of automated morphometry of the mouse brain in the literature to date use large-deformation diffeomorphic metric mapping (commonly called LDDMM) [43] or another TBM method (e.g. 45). In some cases, such as in the case of lesions in otherwise homogeneous areas, high-resolution non-linear image registration methods have an unpredictable response. Image similarity metrics will favour shrinkage of the lesion, but regularisation schemes will oppose tissue shrinking to disappear. For techniques based on determining shape differences, these signal changes can be confounding. Whether they are detected or not will be dependent on the regularisation scheme used. This idea is illustrated in Fig. 9, which shows an *in vivo* image of a mouse brain following an induced lesion taken from a study in our imaging centre.

Two relevant effects of this lesion for the purposes of this discussion are in the anterodorsal caudate putamen (Fig. 9A-C) at the external capsule and in a medial thalamic nucleus (Fig. 9D-F). In our imaging protocol, both areas show marked hypointensity. Maps of GM (Fig. 9A,D) of these sections show a reduction in observed GM probability as would be expected with the change in signal intensity. The Jacobian maps (Fig. 9C, F), as would be analysed in a TBM-like analysis show heterogeneous responses: with large “volume” increases for the striatal lesion and a “volume” decrease for the thalamic lesion.

We do not suggest that TBM studies are inappropriate in the mouse brain but we wish to highlight that in some circumstances VBM-style analyses are more readily interpreted. For studies where shape changes are a particular goal, however, a TBM-like study may still be more relevant. It is not our intention to advocate VBM over other techniques in general but we hope that the validation and results in this paper will draw attention to the technique as a useful alternative to DBM/TBM studies where appropriate.

## 5 Conclusion

We have shown that VBM can be used in the living mouse brain using the R6/2 model of HD. The differences between WT and R6/2 transgenic mice not only fit well with our previous ex vivo study using a larger dataset but also with studies of similar mouse models that used larger group sizes with more demanding techniques [44], [45].

The findings of our previous studies [13], [14] in which we used a large number of brains (n = 89) have been demonstrated with much smaller groups, namely 6 vs. 6. Interestingly, the majority of the changes that we identified using ex vivo, high resolution scans can still be seen in comparatively lower resolution scans even though the animals were one month younger than those used previously. The atrophy seen in the later stages of disease is already detectable four weeks before the mice reach end stage. The demonstration that the technique can detect these differences with small groups suggests this technique has good potential for evaluating potential treatments or therapeutic strategies when applied to cohorts of mice.

We have shown that, in common with the human brain, residuals from fitted models to GM maps deviate from normality. However, we also showed that the deviation has no real effect on the rate of false positives obtained or their distribution across the brain. VBM can be performed quickly with readily available open-source tools templates and offers a translational approach for studies comparable with human approaches that dramatically improve throughput and detection sensitivity over manual volumetric methods.

Software for the SPM package, templates and a full guide can be downloaded from the SPM plugin website (http://fil.ion.ucl.ac.uk/spm).
